# Assessment of Bacterial Community Structure, Associated Functional Role, and Water Health in Full-Scale Municipal Wastewater Treatment Plants

**DOI:** 10.3390/toxics13010003

**Published:** 2024-12-24

**Authors:** Inderjeet Tyagi, Kaomud Tyagi, Faheem Ahamad, Rakesh Bhutiani, Vikas Kumar

**Affiliations:** 1 Centre for DNA Taxonomy, Molecular Systematics Division, Zoological Survey of India, Kolkata 700053, West Bengal, India; kumud.tyagi5@gmail.com; 2 Department of Environmental Science, Keral Verma Subharti College of Science (KVSCOS), Swami Vivekanand Subharti University, Meerut 250005, Uttar Pradesh, India; faheem.ahamad170390@gmail.com; 3 Department of Environmental Science, Gurukul Kangri (Deemed to be University), Hardwar 249404, Uttrakhand, India; rbhutiani@gmail.com

**Keywords:** wastewater treatment plants, bacterial diversity, functional annotations, environmental variables, correlation, sustainable development goals

## Abstract

The present study collected wastewater samples from fourteen (14) full-scale wastewater treatment plants (WWTPs) at different treatment stages, namely, primary, secondary, and tertiary, to understand the impact of WWTP processes on the bacterial community structure, their role, and their correlation with environmental variables (water quality parameters). The findings showed that the bacterial communities in the primary, secondary, and tertiary treatment stages are more or less similar. They are made up of 42 phyla, 84 classes, 154 orders, 212 families, and 268 genera. *Proteobacteria*, *Bacteroidetes*, *Cloacimonetes*, *Firmicutes*, *Euryarchaeota*, *Verrucomicrobia*, *Cyanobacteria*, *Desulfomicrobium*, *Thauera*, *Zavarzinia*, and *Nitrospirae*, among others, dominated the bacterial community structure in all treatment stages. The biochemical oxygen demand was 7–12 times, chemical oxygen demand (COD) was 6 times, and total suspended solids (TSS) was 3.5 times higher in the wastewater than what the Central Pollution Control Board (CPCB) in New Delhi, India, allows as standard discharge. The correlation analysis using the Pearson r matrix and canonical correspondence analysis (CCA) also confirmed the fact that these water quality parameters (especially BOD and COD) play a pivotal role in deciphering the community structure in WWTPs.

## 1. Introduction

Annually, the world produces around 380 billion cubic meters of wastewater [1]. Only 52% of this amount undergoes treatment using existing infrastructure such as WWTPs, sewage treatment plants (STPs), effluent treatment plants (ETPs), constructed wetlands, etc., leaving the remaining 48% untreated for release into aquatic environments [2]. South and Southeast Asia, particularly densely populated areas like India, Pakistan, Malaysia, Indonesia, and China, discharge significant volumes of untreated wastewater into the environment, making these regions hotspots for untreated wastewater [2].

India, a developing country, is currently undergoing industrialization and urbanization at an exponential rate. Although these processes present numerous economic development potentials, they also impose significant anthropogenic stress on the natural resources, particularly water [3,4]. As a result, several harmful toxic and emerging pollutants have entered the aquatic ecosystem, thereby causing water pollution [4]. The 35 states of India collectively produced a total of 72,368 million liters per day (MLD) of sewage wastewater. Only 20,236 MLD (approximately 28%) of this wastewater underwent treatment, leaving the remaining 72% untreated and containing pollutants such as heavy metals, dyes, pesticides, persistent organic pollutants (POPs), polycyclic aromatic hydrocarbons (PAHs), endocrine-disrupting chemicals (EDCs), pathogens, antibiotic resistance genes (ARGs), and antibiotic-resistant microorganisms (AMRs) to discharge into nearby water bodies [5]. Untreated wastewater, a ubiquitous microecosystem, serves as a source–sink combination for numerous microorganisms and can provide valuable insights into ambient bacteria, the human gut, and complex issues such as antibiotic resistance [6,7,8]. Furthermore, it significantly negatively impacts both ecological and environmental health [4]. This leads to changes in the structure and function of microbial communities, eutrophication, and dysfunction of the ecosystem, particularly in relation to priority and emerging contaminants [9,10]. These contaminants undergo bioaccumulation and are then transferred to humans and aquatic animals through various food chains, leading to detrimental effects on their health [11].

Multiple global studies have been conducted to better understand the structure of microbial diversity in WWTPs [12,13,14,15,16,17,18,19]. Dueholm et al. undertook the most comprehensive effort to document the bacterial community structure in 740 WWTPs, and their findings confirmed the existence of a complex network of different bacteria that plays a key role in the biological treatment of wastewater and the maintenance of biogeochemical cycles [12]. Furthermore, they recorded the existence of 1530 species from 966 genera, further classifying them according to their functional roles like nitrification, polyphosphate-accumulating organisms (PAOs), glycogen-accumulating organisms (GAOs), and more [12].

Cao et al. elucidated the microbial diversity in eight Chinese WWTPs, taking into account the V4–V5 regions of target-based 16S rRNA amplicon-based genes as well as their functional roles in biological wastewater treatment and biogeochemical cycles [13]. The obtained results confirmed the presence of 51 bacterial phyla, subsequently taxonomically classified into 24 predominant genera. Based on the functional potential, these 24 genera were categorically classified as denitrifiers, anaerobic fermentation bacteria (AFB), organic degrading bacteria (ODB), PAOs, and nitrite-oxidizing bacteria (NOB). In addition, Chen et al. examined the composition of bacterial communities in eleven (11) full-scale WWTPs that used the same treatment method but treated diverse types of wastewater [13]. They focused on the V3-V4 region of the 16S rRNA gene [14]. The acquired results confirmed the existence of bacterial diversity accounting for functional annotations such as ammonia-oxidizing bacteria (AOB), heterotrophic bacteria, PAOs, and others [14]. In their research, Gao et al. looked at the variety of bacteria that live in industrial WWTPs. They specifically looked at biodegradation genes (BDGs) and organic degradation genes (ODGs). They utilized whole metagenomics sequencing (WMS) for their analysis [15].

In the Indian context, there have been limited efforts to investigate the microbial diversity in WWTPs using advanced omics-based techniques like whole-genome sequencing (WGS) or targeted sequencing of the 16S rRNA gene [8,20]. Gupta et al. investigated the impact of heavy metals on the development of antibiotic resistance genes (ARGs) and mobile genetic elements (MGEs) in two STPs located in New Delhi, the capital of India [20]. The results revealed the inefficiency of the current sewage treatment system in terms of the complete elimination of detrimental pollutants such as heavy metals and emerging pollutants like ARGs [20]. The primary constraint associated with this study was the lack of data pertaining to the structure and functional importance of the microbial population involved in the wastewater treatment. Furthermore, the influence of wastewater treatment techniques on the proliferation or eradication of microorganisms also was not studied. In contrast, Singh et al. conducted a study on the microbiome found in Indian sewage. They confirmed the existence of distinct microbial communities, including harmful microorganisms, in the raw sewage water collected from around 47 diverse locations that represented various biogeographical conditions and demographics [8]. The results obtained confirmed the presence of 52 phyla, with *Proteobacteria* being the most prevalent. These phyla were further taxonomically divided into 160 differentially abundant bacterial genera. Furthermore, pathogenic genera such as *Pseudomonas*, *Aeromonas*, *Escherichia*/*Shigella*, *Acinetobacter*, *Aeromicrobium*, and others were also observed in the sewage samples from India [8]. Until now, this is one of India’s largest and most significant efforts that reveals the structure of the microbial community in untreated sewage water (raw sewage). However, a limitation of this study was the lack of exploration into the impact of wastewater treatment technologies (primary, secondary, and tertiary) on the structure of the microbial community. Moreover, in the present study, variables such as diverse geographical locations (with varying temperatures) and demography (including cultural and dietary habits) have a substantial impact on the growth and spread of the microbial community derived from untreated sewage water.

Keeping in view the need of the hour, we collected wastewater from fourteen (14) fully operational wastewater treatment plants (WWTPs) using primary, secondary, and tertiary treatment methods. The main objective was to investigate the composition of the bacterial communities and their predicted functions (such as biological remediation, biosynthesis, and metabolic pathways) in the WWTPs. More specifically, the current study will provide answers as follows: (1) It will investigate the structure of bacterial communities in wastewater treatment plants (WWTPs) and their predicted functional roles. (2) It will investigate the water quality of the WWTPs in terms of physicochemical parameters and their correlation with the abundant bacterial taxa. Lastly, it will provide recommendations to enhance existing wastewater treatment technologies in order to align with the sustainable development goals (SDGs), especially goals 3 and 6. Goal 3 (Good Health and Well-Being) places significant emphasis on objective 3.9, which focuses on the health consequences caused by pollutants and contaminants as well as on reducing the deaths and illnesses resulting from these toxic contaminants in the air, water, and soil by 2030. On the other hand, goal 6 (Clean Water and Sanitation) places significant emphasis on objective 6.3, which aims to improve water quality and promote the recycling and safe reuse of water. This can be achieved by reducing pollution and enhancing the performance and capacity of WWTPs/STPs, thereby reducing the volume of untreated wastewater and promoting recycling and safe use globally.

## 2. Materials and Methods

### 2.1. WWTPs’ Structure

In India, the primary treatment process of WWTPs consists of screening/microstraining, grit removal, and primary clarification [21]. It removes algae, plankton, and large solid waste from the raw water (influent) ([21], https://www.niti.gov.in/sites/default/files/2022-09/Waste-Water-A4_20092022.pdf Accessed on 15 April 2024). After the primary treatment, the wastewater undergoes a secondary treatment process, its main components being aeration, coagulation, flocculation, sedimentation, and filtration [21]. Aeration involves the transfer of oxygen to the aerobic biomass, which is responsible for converting organic matter into biomass. Thus, introducing air or other gases into the water eliminates volatile substances such as dissolved gases, volatile organic compounds, and various aromatic compounds that cause taste and odor issues [21]. Coagulation and flocculation refer to the use of a chemical or physical method to mix a coagulating chemical with a stream, which is then gently stirred. Lastly, the previous processes of coagulation, flocculation, and sedimentation commonly support filtration, the most reliable process in wastewater treatment, to ensure effective wastewater treatment.

**Figure 1:**
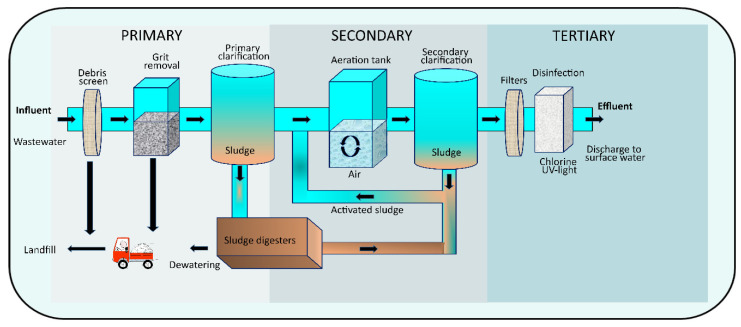
Schematic presentation of full-scale WWTPs representing treatment stages, namely, primary, secondary, and tertiary.

The tertiary treatment process involves the application of disinfectants, with chlorine being the most widely used disinfectant [21]. Although chlorine is considered the most significant breakthrough in the water-treatment process, researchers worldwide have recently focused on the disinfection by-products (DBPs) generated by chlorine to explore alternative disinfectants [21]. The schematic outline of the WWTPs arrangement can be better understood from Figure 1.

### 2.2. Sample Collection

Forty-two (n = 42) wastewater composite samples (5 L each) were obtained from the primary, secondary, and tertiary treatment units of fourteen (14) full-scale WWTPs situated in India and located under similar geographical conditions (precise locations masked due to confidentiality clause). The samples were collected in July 2019 after obtaining official permission from the competent state authorities. Collectively, these can process approximately 179 MLD of household wastewater sourced from nearby residential colonies while running at full capacity, but while sampling, they had loads of only 112.2 MLD (Table S1). The wastewater samples were separated into two portions. One portion, consisting of 4 L each of primary, secondary, and tertiary treatment samples, was used for environmental DNA (e-DNA) analysis. The remaining 1 L was utilized to assess the physicochemical parameters in order to evaluate the water quality and health of the WWTPs. The wastewater samples collected for e-DNA study were transported to our laboratory at ZSI, Kolkata in airtight HDPE jars, maintaining a cold chain temperature of approximately 4 °C through portable coolers. The transportation was completed within two days of collection. In order to elucidate the impact of wastewater treatment technologies on the bacterial diversity structure and to enhance the accuracy of the results, the methodology was designed in such a way as to rule out the influence of elements such as geographical location and demography. Additionally, these WWTPs treat nearly comparable types of wastewater, specifically, domestic sewage, and do not include any industrial or hospital liquid waste, to the best of our understanding. The lack of replication in the present study due to limited infrastructural resources is the major limitation of the present work. Additional limitations are the one-time sampling of these WWTPs and the failure to account for seasonal variations, which could significantly impact the bacterial diversity composition and dynamics due to dilution.

### 2.3. Water Quality Parameters (Physicochemical Characteristics)

In order to assess the water quality in wastewater treatment plants (WWTPs), the Central Pollution Control Board (CPCB) in India recommends the monitoring of parameters such as pH, total suspended solids (TSS), BOD measured at 5 days, COD, nitrate (NO_3_^−^), sulfate (SO_4_^2−^), BOD/COD ratio, etc. Out of these parameters, pH was measured on-site using a portable water monitoring kit (HI9829, Hanna Instruments, Romania). The remaining parameters, including TSS, BOD, COD, nitrate (NO_3_^−^), and sulfates (SO_4_^2−^), were analyzed in the laboratory using the standard protocol recommended by the American Public Health Association [22] and the CPCB, New Delhi [23].

### 2.4. Wastewater Filtration, DNA Extraction, and Target-Based 16S rRNA Amplicon Sequencing

A mixed cellulose esters (MCE) hydrophilic membrane filter (MF-Millipore, Merck, India) with a pore size of 0.22 µm was used to filter the 42 samples of wastewater. The filtration process continued until the membrane filter paper became clogged. Approximately 2 L of wastewater was filtered on one membrane filter paper, while the remaining 2 L was filtered on another membrane filter paper as a replicate for the separate isolation of DNA. The 0.22 µm filter papers (n = 84) were initially cut into small fragments using sterile scissors and subsequently utilized for DNA extraction. The DNeasy PowerWater Kit (Qiagen, Hilden, Germany, Cat No. 14900-100-NF) was employed for this purpose, following the standard methodology provided in their catalogs. The DNA obtained from duplicate samples was thereafter combined with the main samples and stored at a temperature of −20 °C until the sequencing process. The qualitative and quantitative assessment of the extracted DNA was carried out using agarose gel electrophoresis (Cell BioScience Alphamager MINI, Fisher, UK) and the Qubit 2.0 Fluorometer (Q32866, Thermo Fisher, Kolkata, India).

We used the primer sets 515F-Y (5′-GTGYCAGCMGCCGCGGTAA-3′) and 926R (5′-CCGYCAATTYMTTTRAGTTT-3′) for the amplification of the hypervariable V4–V5 region of 16S rRNA following the PCR conditions previously mentioned [13,24]. We purified the amplified PCR products using the Agencourt AMPure XP purification system (Beckman Coulter, Brea, CA, USA). The qualified constructed Nextera library was then sequenced using the Illumina MiSeq platform, considering paired-end 2 × 300 bp chemistry. The generated raw reads were submitted to the National Center for Biotechnology Information (NCBI) database under the Bio Project ID: PRJNA1011483 with accession number SAMN37220753-SAMN37220795.

### 2.5. Data Processing and Bioinformatics

We analyzed the raw data from the sequencing platform using the QIIME2-2019.10 version of the Quantitative Insights into Microbial Ecology software [25]. The generated raw sequences underwent demultiplexing, quality filtering, trimming, denoising, chimera removal, and merging using the DADA2 pipeline [26]. A total of 17,021 amplicon sequence variables (ASVs) were generated after processing a total of 5,516,140 clean reads, with an average of 128,282 reads per sample. The ASVs obtained were taxonomically classified at the genus level using the QIIME2 feature classifier. This classification was performed with a 99% similarity threshold against the SILVA database (version 138). The outcomes, namely ASVs, taxonomy classifiers, and metadata files, were used for further downstream analysis such as taxonomic profiling and alpha (α-) and beta (β-) diversity in Microbiome Analyst using the marker data profiling (MDP) module [27,28,29]. Under this module, singleton ASVs were removed, resulting in only 7446 ASVs for further downstream analysis. Furthermore, the data were filtered considering default parameters such as low-count filter with minimum count of 4 and prevalence in samples of 20% with a low-variance filter with percentage to remove 10% based on inter-quartile range. Filtering resulted in the removal of 6433 low-abundance features based on their prevalence. For taxonomic classification, we used filtered data, while for calculating species richness (alpha (α-) and beta (β-) diversity), the original data were used. For studying alpha (α-) diversity, we used T-tests and ANOVA to look at richness estimators like Chao1, observed, and diversity estimators like Shannon and Simpson. For studying beta (β-) diversity, we used PCoA and NMDS plots with the Bray–Curtis index as a distance matrix and PERMANOVA to look at the differences in bacterial diversity in three treatment stages of WWTPs: primary, secondary, and tertiary. For the Venn diagram, we used EVenn software. The Origin 2024b software developed by OriginLab Corporation, Northampton, MA, USA was used for statistical analysis (Pearson correlation) between bacterial community composition and environmental variables.

### 2.6. Predicted Functional and Metabolic Potential

Based on the amplicon dataset, the predicted functional and metabolic role of bacterial community structure in the degradation of organic impurities, regulating nutrient cycles, biosynthesis pathways, etc., in WWTPs was analyzed using PICRUSt2 [30]. Based on the nearest sequenced taxon index (NSTI), the functional role of taxonomically classified 16S rRNA sequences was allocated, which was later followed by the reconstruction of metabolic roles using Kyoto Encyclopedia of Genes and Genomes (KEGG) databases [31,32,33]. The functional predictions were created using the predict_metagenomes.py script, and the obtained KEGG pathway metadata were combined at the third (lowest) functional category using the PICRUSt2 pipeline full annotations. We used the Statistical Analysis of Metagenomics Profiles (STAMP) standalone tool [34] for statistical verifications and corrections. The PICRUSt2 analysis has several constraints and limitations such as dependence on reference genomes, functional conservation assumption, limitations in detecting novel functions, selection of marker genes, relative accuracy to shotgun, computation requirements, etc., and these limitations play a pivotal role in understanding the utility and interpretation of the work. More details about the same may be obtained from the Supplementary Materials.

## 3. Results and Discussions

### 3.1. Water Quality Parameters

An analysis was conducted on the water quality parameters, such as pH, BOD, COD, BOD/COD ratio, TSS, nitrate (NO_3_^−^), and sulfate (SO_4_^2−^), of several wastewater samples collected from different treatment stages, namely primary, secondary, and tertiary. The results obtained are summarized in Table 1. The pH of the samples ranges from slightly acidic (6.5) to slightly alkaline (8.8) across the treatment groups. These findings fall within the acceptable range of pH levels for discharge, which is between 5.5 and 9.0 (Table 1).

The BOD levels in the samples collected from the primary, secondary, and tertiary treatment groups range from 76 to 130 mg/L, 75 to 135 mg/L, and 75 to 120 mg/L, respectively. The COD values for the primary, secondary, and tertiary treatment groups fall within the ranges of 325–380 mg/L, 300–370 mg/L, and 280–365 mg/L, respectively. The levels of BOD and COD in the released effluents (tertiary treated sample) are seven to twelve times higher for BOD and about six times higher for COD in comparison with the standard limits allowed by the CPCB for Indian wastewater treatment plants (10 mg/L for BOD and 50 mg/L for COD) (Table 1). The high concentration of BOD and COD in the wastewater samples indicates the presence of high organic matter, thereby providing significant environments for the proliferation of microbial communities. Furthermore, the high concentration of BOD and COD suggests that the selected WWTPs are not effectively treating the wastewater samples; consequently, the impact of different treatment stages (primary, secondary, and tertiary) on the removal of BOD and COD from wastewater is minimal. The low variation in concentration of BOD and COD from the secondary to the tertiary treatment group wastewater samples can be attributed to the fact that the disinfection process (chlorination) does not affect these two water quality indicators, as previously demonstrated [35]. On the other hand, the low variation in concentrations of BOD and COD from the primary to the secondary treatment stage wastewater samples signifies the ineffective functioning of the WWTPs.

The BOD/COD ratio, a potential indicator of the impact of organic matter-containing wastewater in environmental components such as WWTPs [36], varies in the range of 0.23–0.37 for the primary, secondary, and tertiary treatment groups’ wastewater samples (Table 1). A low BOD/COD ratio indicates the presence of toxic components (non-biodegradable salts and hazardous wastes) and falls under the category of heavily polluted water, where biodegradation can be achieved only with the application of physical and chemical wastewater treatment technologies [36].

The TSS for the wastewater samples lies in the range of 82.5 to 159 mg/L, 78.1 to 141.2 mg/L, and 71 to 120.3 mg/L in the primary, secondary, and tertiary treatment groups, respectively. It is approximately three and a half (3.5) times higher than the standard discharge norms (20 mg/L) (Table 1). The high level of TSS in the wastewater samples shows that the treated wastewater has poor water quality, with many inorganic substances (like silt, fine sand, and aquatic minerals) and organic substances (like detrital particles made up of carbohydrates, proteins, and lipids) [37,38].

The nitrate (NO_3_^−^) concentration in the wastewater samples ranges from 10.2 to 25.6 mg/L (primary), 8.2 to 22.3 mg/L (secondary), and 6.3 to 16.5 mg/L (tertiary). These concentrations are well within the Indian standard permissible limits (50 mg/L) (Table 1). The presence of nitrate in water supplies has emerged as a significant environmental issue. Excess concentration of nitrate in water and wastewater results in river eutrophication, thereby affecting the water quality [39]. Moreover, it can undergo reduction to form nitrite (NO_2_^−^), which poses severe detrimental impacts on human health, such as liver damage, methemoglobinemia, and possibly leading to the development of cancer [40].

The sulfate (SO₄²⁻) concentration in the wastewater samples ranges from 12 to 44 mg/L (primary), 15 to 45 mg/L (secondary), and 15 to 48 mg/L. The concentration of sulfate is within the standard permissible limits (<150 mg/L) (Table 1). High concentrations of sulfates generate precipitates on the beds of streams that obstruct the areas necessary for the inhabitation and reproduction of aquatic species [41]. It generally does not pose any serious detrimental impact on human health. However, low concentrations of sulfates in drinking water (250 to 500 mg/L) might result in an unpleasant taste [42]. Furthermore, higher concentrations of sulfates (>1000 mg/L) can lead to illnesses such as diarrhea [43]. Furthermore, higher concentrations of sulfates (>1000 mg/L) can disrupt the aquatic ecosystem by providing nourishing media for algal blooms, which can alter food webs and disturb the ecological balance. Due to their digestive metabolism, which converts sulfates into poisonous hydrogen sulfide (H_2_S), ruminant animals are at high risk from sulfates [44].

### 3.2. Sequences, Rarefaction, and ASV Distribution

A total of 5,516,140 high-quality sequences were acquired via targeted amplicon sequencing (16S rRNA). The sequence lengths ranged from a maximum of 482,802 to a minimum of 61,617, with an average read count per sample of 128,282. The high-quality sequences were categorized into 17,021 ASVs. After removing singletons, a total of 7446 ASVs were retrieved for downstream analysis in Microbiome Analyst.

The rarefaction curves (Figure S1) for the full-scale wastewater treatment samples showed that the sequencing depth was good enough to look at the bacterial community structure and species diversity at all stages of treatment, including primary, secondary, and tertiary.

**Figure 2:**
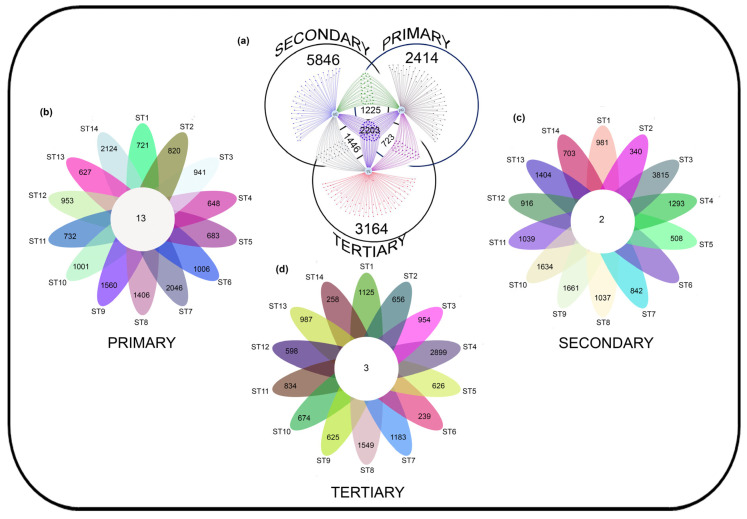
Venn diagram of obtained ASVs. (**a**) Cumulative ASVs shared and unique in different treatment stages (primary, secondary, and tertiary); (**b**) shared and unique ASVs between wastewater samples obtained from the primary treatment stage; (**c**) shared and unique ASVs between wastewater samples obtained from the secondary treatment stage; (**d**) shared and unique ASVs between wastewater samples obtained from the tertiary treatment stage.

The Venn analysis revealed that the primary, secondary, and tertiary treatment stages shared 2203 core ASVs, which accounted for 12.94% of the total 17,021 ASVs. In terms of unique ASVs, the primary, secondary, and tertiary treatment stages had 14.18%, 30.82%, and 18.58% ASVs, respectively (Figure 2a). Furthermore, a comparison of the wastewater samples from the primary treatment group revealed that the 14 WWTPs shared only 13 core ASVs. However, there was a range of 627 to 2124 ASVs that were considered unique (as shown in Figure 2b). Similarly, in the secondary treatment group, the 14 WWTPs shared only 3 core ASVs, while a range of 239 to 2899 ASVs were considered unique (as shown in Figure 2c). The tertiary treatment group shared only 2 core ASVs and classified the remaining ASVs, ranging from 340 to 3815, as unique (Figure 2d).

### 3.3. Characterization of Bacterial Diversity

Based on the amplicon sequencing, these full-scale WWTPs contribute 42 phyla, 84 classes, 154 orders, 212 families, and 268 genera to the bacterial community structure in the primary, secondary, and tertiary treatment stages.

#### 3.3.1. Bacterial Diversity Composition and Community Structure

**Figure 3:**
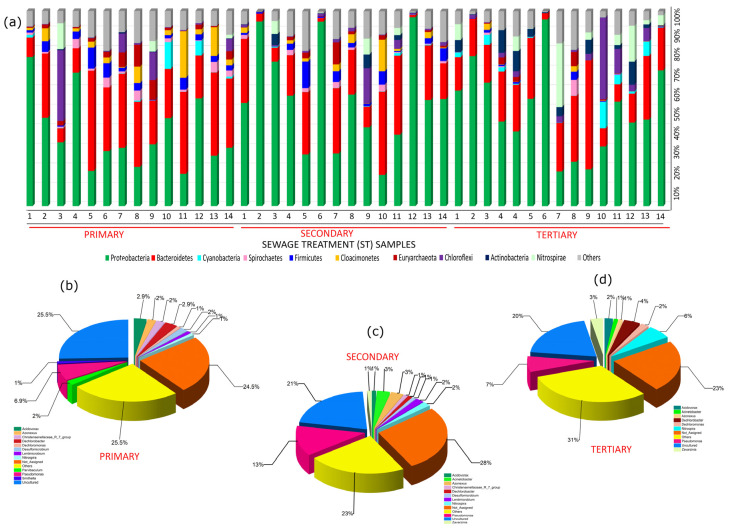
Composition and structure of bacterial diversity in different treatment stages (primary, secondary, and tertiary) (**a**) at phylum level (data representing top 10% abundant phyla); Pie chart showing genus abundance in (**b**) primary; (**c**) secondary; (**d**) tertiary (data representing top 20% abundant genera, low-abundance genera are included under the heading “Others”).

Phyla such as Proteobacteria and Bacteroidetes significantly dominated the bacterial diversity abundance in all treatment groups, including primary, secondary, and tertiary (Figure 3a). The obtained results showed an increase in the average abundance of Proteobacteria members from 39 to 53% when they transitioned from the primary to the tertiary treatment group. Further, the genus belonging to the class Gammaproteobacteria, with an average abundance of 26–39%, followed by Alphaproteobacteria (5–30%) and Deltaproteobacteria (2–6%), dominated the bacterial diversity in the dataset. The findings were in line with [8,13], where members of the phylum Proteobacteria dominated the bacterial diversity in, respectively, the full-scale WWTPs and Indian sewage. Globally, WWTPs/STPs have shown similar trends in the abundance of phylum Proteobacteria [12,17,45,46,47,48,49,50,51]. The high concentration of water quality parameters like TSS, BOD, and COD may contribute to the high abundance of phylum Proteobacteria, as their members play a significant role in various cycles such as the global carbon, nitrogen, phosphorus, and sulfur cycles [13,52,53]. Additionally, the members play an effective role in amino acid metabolism and degradation of complex organic compounds [54,55,56,57]. At the genus level, approximately 23–28% of sequences read were characterized as “not assigned”, ~20–26% as “uncultured bacteria”, and the genera that were smaller in abundance were shown as others (~23–31%) (Figure 3b). Further, genera such as *Pseudomonas*, *Azonexus*, *Acinetobacter*, *Dechlorobacter*, *Dechloromonas*, *Desulfomicrobium*, *Thauera*, *Zavarzinia*, etc. majorly contributed toward the abundance of the phylum Proteobacteria in different treatment groups. The genera *Pseudomonas* and *Thauera* are well known for their pivotal role in WWTPs in denitrification–desulfurization systems, where they oxidize sulfide to elemental sulfur through denitrification [58,59]. The literature suggests that the genus Pseudomonas is the main autotrophic denitrifiers in WWTPs [59]. On the other hand, the genus *Thauera* can lower nitrate-nitrogen (NO_3_^−^-N) and nitrite-nitrogen (NO_2_^−^-N), denitrify phosphorous, and break down complex aromatic compounds [3,59,60,61]. The genus *Acinetobacter* is widely known as a phosphorous-accumulating organism (PAO), and it plays a significant role in the removal of phosphorous from the WWTPs [62]. *Desulfomicrobium*, a sulfate-reducing bacterium, may also be able to use ethanol (CH₃CH₂OH) as an electron donor and lower sulfates in WWPTs [63]. Further, it also has the potential to consume residual sulfur and complex organic substances formed due to other bacterial fermentations [63,64].

Members of the genus *Azonexus*, belonging to the family Rhodocyclaceae, are classified as chemoorganoheterotrophs, aerobic and microaerophilic microorganisms that play a significant role in terms of nitrogen metabolism and fixation [65]. To date, only three species, namely *Azonexus fungiphilus*, *Azonexus caeni*, and *Azonexus hydrophilus*, are known to the world; they are reported from Black Sclerotia of Basidiomycete, rice fields, Pakistan; sewage sludge, South Korea; and freshwater springs, located at Hsinchu County, Taiwan and Inha University, South Korea, respectively. Among the three, two species, namely *Azonexus caeni* and *Azonexus hydrophilus*, use nitrate as an electron acceptor [65,66]; thus, it can be inferred that one or both of these species may be present in our dataset and have a potential role in nitrogen metabolism and fixation [66]. On the other hand, genus *Dechlorobacter* and *Dechloromonas*, also belonging to the family Rhodocyclaceae, are classified as chemoorganoheterotrophs with a strictly respiratory metabolism. They oxidize acetate with O_2_, chlorate, perchlorate, or nitrate [65]. They are cumulatively termed “chlorate reducers”, and the species belonging to these genera are known for the degradation of complex organic aromatic compounds such as chlorobenzoate, toluene, and xylene, using perchlorate as an electron acceptor [65].

The genus *Zavarzinia* has a potential role in the degradation of emerging contaminants such as pharmaceutical impurities [67]. The authors concluded through genome reconstruction analysis that *Zavarzinia* possesses encoded genes known for the biodegradation of xenobiotic compounds (benzoate/aminobenzoate), aromatic compounds (toluene, xylene, ethyl-benzene), and polycyclic aromatic hydrocarbons (naphthalene), among others [67].

The average abundance of members of phylum Bacteroidetes in the secondary and tertiary treatment groups decreases substantially by ~8–9% in comparison with that of the wastewater samples obtained from the primary treatment group. Krieg et al. [68] classified the phylum Bacteroidetes into several classes, including Bacteroidia, Cytophagia, Flavobacteriia, and Sphingobacteriia, but only Bacteroidia was present, and it dominated the bacterial diversity. The high abundance of phylum Bacteroidetes may be attributed to the high concentration of organic matter (high BOD and COD), as the members belonging to this phylum are generally responsible for the decomposition of complex, high-molecular-weight compounds, namely carbohydrates and polymers, into simpler compounds, such as lactate and ethanol, that can be utilized for the metabolic activities of the different microbial communities [53,69,70]. Other than Proteobacteria and Bacteroidetes, phyla with an average abundance such as Cloacimonetes (5%), Firmicutes (4%), the Archaeal representative Euryarchaeota (3%), Chloroflexi (2%), Cyanobacteria (2%), Verrucomicrobia (2%), Nitrospirae (1%), etc., dominated the bacterial diversity in the wastewater samples obtained from the primary treatment group.

The members of phylum Cloacimonetes were previously reported from the anaerobic digesters of the WWTPs located in Marrakech, Morocco [71], and they have a great metabolic functional potential to degrade cellulose and amino acids in syntrophic propionate oxidation and low-molecular-weight metabolic products such as acetate, carbon dioxide (CO_2_), and hydrogen (H_2_), respectively [71]. Based on their metabolic potential, generally, they are present in close association with H_2_-consuming or acetate-utilizing archaeal members [71,72,73].

On the other hand, the members of phylum Firmicutes, previously reported from different wastewater treatment plants located across the globe [11,13,14,69], are well known for their activities related to methane degradation [14] and the degradation/decomposition of pollutants [74]. Moreover, members of the phylum Firmicutes have the ability to form spores to withstand extreme conditions, which is one of the unique reasons for this phylum’s widespread presence in various WWTPs worldwide [75,76].

The ubiquitous presence of Archaea under the phylum Euryarchaeota was previously reported from the anaerobic digesters of WWTPs [77]. The phylum Euryarchaeota is classified into six well-established orders, namely, Methanobacteriales, Methanococcales, Methanomicrobiales, Methanosarcinales, Methanopyrales, Methanocellales, and Methanomassiliicoccales (as proposed) [77]. As the names suggest, the members of this phylum are generally called methanogens, and they depend on limited substrates such as H_2_, CO_2_, methyl group-containing compounds, and acetate for their metabolic activities. They have great functional potential in the anaerobic digestion of sludge through generating the value-added product methane as a by-product [77].

Researchers previously reported the presence of the phylum Cyanobacteria in sludge samples from WWTPs in various Indian districts and the world’s largest sewage-fed fish farms (the EKWs) in Kolkata, India [8,78]. The literary evidence suggests that members of this phylum play significant roles in nitrogen fixation by converting it to nitric oxide (NO_3_), inorganic carbon fixation by converting it to methane (CH_4_), the decomposition of complex organic molecules, and the generation of bioenergy [8,78,79]. Moreover, the high abundance of phylum Cyanobacteria poses a negative impact on the wastewater treatment capacity of wetlands by disrupting their carbon metabolic balance [8].

The members of phylum Verrucomicrobia are generally present in diverse environments, ranging from freshwater ecosystems to animal guts [80]. The global abundance of this phylum in the freshwater and marine water environment is known for the degradation and hydrolysis of polysaccharides [80]. Additionally, recent developments in terms of extremophile Verrucomicrobial methanotrophs from acidic geothermal environments open up new scopes in terms of the metabolic functional potential [81]. The literary evidence suggests that Verrucomicrobial methanotrophs possess a significant metabolic functional potential to utilize inorganic gases such as CH_4_, H_2_, CO_2_, N_2_, NH_3_, and H_2_S through various enzymatic pathways. As a result, they are considered a significant phylum that plays a crucial role in various biogeochemical cycles and in the remediation of greenhouse gases (GHGs) in geothermal ecosystems [81]. In sewage/WWTPs ecosystems, the presence of the Verrucomicrobia may be attributed to the low pH and abundance of CH₄ or H₂S [81].

Moving from the primary to the secondary treatment group, the average abundance of the phyla Cloacimonetes, Firmicutes, Euryarchaeota, Verrucomicrobia, and Cyanobacteria decreases by 3% for Cloacimonetes and 1% for the rest of the phyla. On the other hand, phyla such as Chloroflexi and Nitrospirae show significant enhancement in terms of average abundance and increase by 4% and 1%, respectively. In addition to this, members of the phylum Actinobacteria cumulatively show an average percentage abundance of 1% after moving from the primary to the secondary treatment group. Previous studies on the Indian sewage microbiome reported the presence of these phyla with low to medium average percentage abundance [8].

Moving from the secondary to the tertiary treatment group revealed similar trends, with phyla such as Nitrospirae, Chloroflexi, and Actinobacteria dominating the bacterial diversity with cumulative average percentage abundances of 6, 5, and 5%, respectively, while members of the phyla Firmicutes and Cloacimonetes were absent from most tertiary treated wastewater samples, and members of the phyla Cyanobacteria, Verrucomicrobia, and Euryarchaeota showed cumulative average percentage abundances of 2, 2, and 1%, respectively.

The members of phylum Chloroflexi were also previously reported from activated sludge samples obtained from different WWTPs, and based on their findings, the authors concluded that this phylum is generally present in the WWTPs designed for remediating nitrogen (N) and phosphorous (P) [82]. Along with that, this phylum’s members are famous for playing a big part in breaking down complex organic polymeric compounds and fermenting carbohydrates into metabolic products that can be used to help other types of bacteria grow [82]. Members of the phylum Chloroflexi also possess the ability to utilize halogenated organic compounds as substrates, acting as electron acceptors for the degradation of chlorocyclohexane (C_6_H_11_Cl) and chlorobenzene (C_6_H_5_Cl) [83].

The members of phylum Nitrospirae are ubiquitously present in WWTPs across the globe [13,53,76,84,85,86] and show comparatively high abundance in the wastewater samples collected from the tertiary treatment group in the current dataset. The members of this phylum are well known for their potential in the fixation or removal of nitrogen from WWTPs through nitrification and denitrification [77]. In terms of genus, *Nitrospira* was found more abundant (5%) in the wastewater samples collected from the tertiary treatment group. The findings obtained are in line with [85], where a high abundance of Nitrospira was observed in the tertiary rotating biological contactors (RBCs) biofilm samples obtained from Guelph WWTP. *Nitrospira* are chemolithoautotrophic nitrite-oxidizing bacteria (NOB) known for catalyzing the second step of nitrification in order to remediate excess nitrogen from the WWTPs [84]. Furthermore, *Nitrospira*, through complete ammonia oxidation (comammox), possess the metabolic capacity to convert cyanate into other metabolites, as previously noted [85].

These narrow shifts in the average percentage abundance of different phyla may be attributed to the treatment process adopted in the secondary and tertiary treatment groups. The application of different strong coagulants, flocculants, and disinfectants in the secondary and tertiary treatment processes may have an antagonistic effect on the abundance of phyla like Cloacimonetes, Firmicutes, Euryarchaeota, Verrucomicrobia, and Cyanobacteria, while it may have a synergistic impact on the abundance of phyla like Nitrospirae, Chloroflexi, and Actinobacteria. Researchers have confirmed the synergistic and antagonistic effects of anaerobic digestion and different coagulants on the abundance of different phyla, including Cloacimonetes [87]. Aside from that, our results suggest that Nitrospirae, Chloroflexi, Actinobacteria, Proteobacteria, and Bacteroidetes members have become resistant to common coagulants, flocculants, and disinfectants that have been used for a long time in many types of wastewater treatment. Further, future studies in this particular direction are required to confirm the resistance of specific phyla against these chemicals.

#### 3.3.2. Diversity Indices

**Figure 4:**
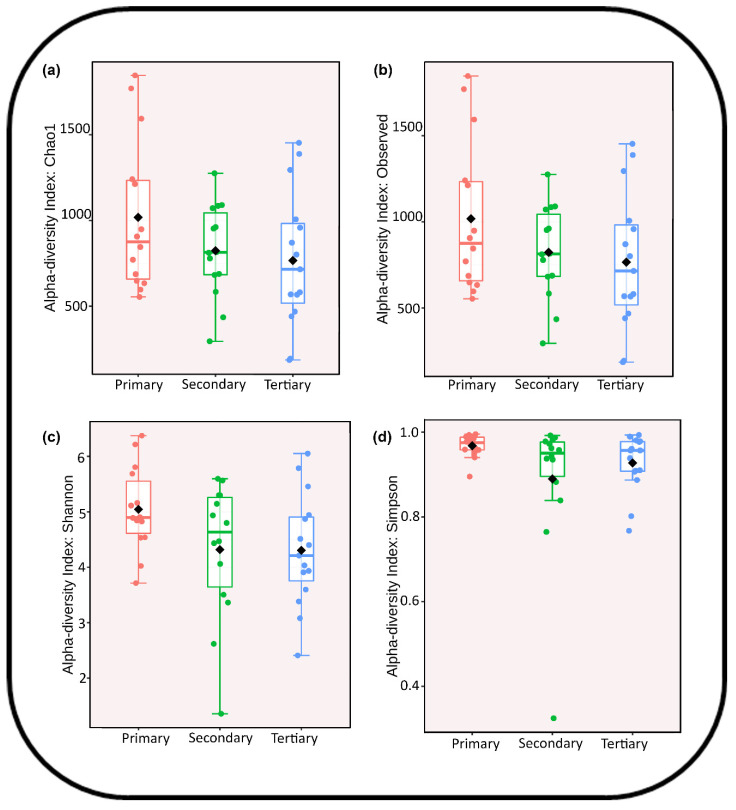
Box-plot showing alpha diversity species richness and evenness indices on raw data in different treatment groups, namely, primary, secondary, and tertiary: (**a**) Chao1; (**b**) Observed; (**c**) Shannon; (**d**) Simpson.

Using diversity measures like Chao1, Observed, Shannon, and Simpson, the alpha (α-) diversity indexes were calculated to examine the evenness and richness of the species in wastewater samples collected from the primary, secondary, and tertiary treatment groups. It was found that the community structure diversity did not change significantly (*p* value < 0.05). However, the wastewater samples from the primary treatment group (1018.21 ± 444.22) were more diverse than those from the secondary (823.35 ± 272.99) and tertiary (766.2 ± 378.07) treatment groups (Figure 4a,b, Table S2). Also, alpha (α-) diversity measures like Shannon and Simpson confirmed that the wastewater samples from the primary treatment group had a higher richness (statistically non-significant *p* value ˂ 0.05) than those from the secondary (Shannon: 4.31 ± 1.23 and Simpson: 0.88 ± 0.17) and tertiary (Shannon: 4.30 ± 0.88 and Simpson: 0.92 ± 0.05) treatment groups (Figure 4c,d, Table S2). The differences in the evenness and richness of the bacterial community structures may be attributed to the load of pollutants (BOD and COD), which is comparatively higher in primary treatment groups than in secondary and tertiary treatment groups. Moreover, the primary treatment group received raw sewage water, which may be one of the reasons for the high richness and evenness of the community structures in the primary treatment group wastewater samples. Furthermore, it is possible to interpret the small variation (0.04 ± 0.12) in the richness and evenness of wastewater samples from the secondary and tertiary treatment groups as evidence that, over time, the diversity of microorganisms, whether pathogenic or non-pathogenic, has grown resistant to commonly used disinfectants, in this case, chlorine.

**Figure 5:**
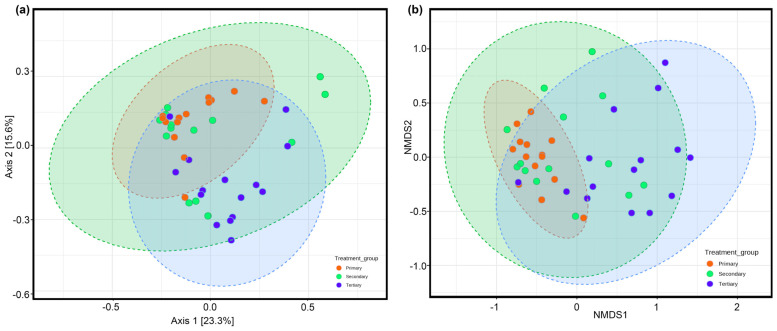
(**a**) Principal coordination analysis (PCoA) of the bacterial community structure based on the Bray–Curtis index method at the genus level present in wastewater samples (F-value: 2.506; R-squared: 0.11135; *p*-value: 0.003). (**b**) Non-Metric Multidimensional Scaling (NMDS) analysis was performed at the genus level (F value: 2.759, R^2^: 0.12, *p* value ˂ 0.05, NMDS stress: 0.13175).

For beta (β-) diversity, the PCoA plots (Figure 5a) at the genus level (F-value: 2.506; R-squared: 0.11135; *p*-value: 0.003) indicated that the bacterial diversity patterns of the wastewater samples obtained from different treatment groups, namely primary, secondary, and tertiary, were closely associated with each other. The low values of PCoA confirmed that the community structures within these treatment groups were more or less similar, with no distinct variation. Furthermore, we can conclude that the wastewater samples from the secondary and tertiary treatment groups exhibit a higher resemblance in terms of bacterial community structures compared with those from primary vs. secondary and primary vs. tertiary. Also, the pairwise PERMANOVA test showed that the differences in the wastewater samples from primary vs. tertiary and secondary vs. tertiary were statistically significant (*p*-value < 0.05, FDR adjusted). On the other hand, the pairwise PERMANOVA analysis of primary vs. secondary treatment groups yielded non-significant variation (*p*-value ˃ 0.05). The findings were in line with those of Kumar et al. (2023), where sewage samples obtained from different geographical locations in India showed similar trends in microbial diversity patterns.

The NMDS plot (F value: 2.759, R^2^: 0.12, *p* value < 0.05, NMDS stress: 0.13175) at the genera level confirmed the results observed in the PCoA ordination plots (Figure 5b). The majority of the wastewater samples from the primary treatment group clustered together at low confidence intervals, while only a few samples from the secondary and tertiary treatment groups significantly overlapped. Furthermore, the clustering of the majority of the wastewater samples from the secondary and tertiary treatment groups revealed a relatively higher degree of similarity in the structures of the bacterial communities.

#### 3.3.3. Correlation Analysis Between Environmental Variables and Bacterial Diversity Composition

**Figure 6:**
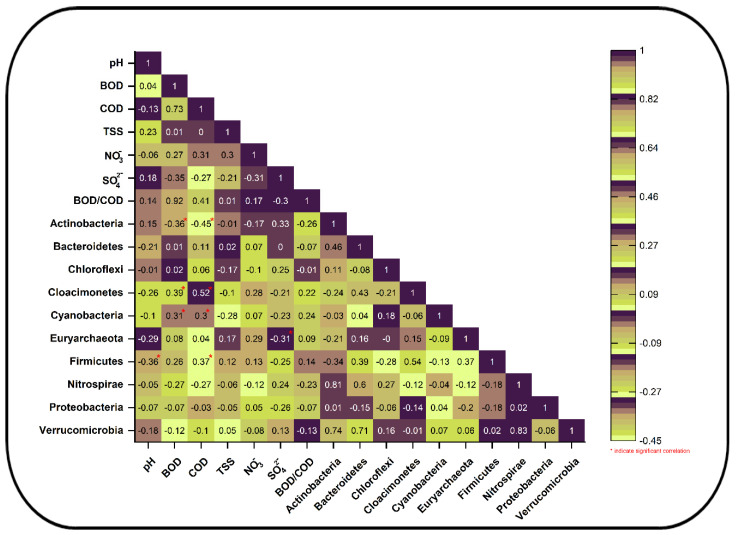
Pearson correlation matrix between environmental variables (water quality parameters) and bacterial phylum abundance. Boxes with values * represent significant correlation between environmental variables and bacterial diversity.

We used a Pearson r correlation matrix to investigate the impact of environmental variables such as pH, BOD, COD, TSS, NO_3_^−^, SO_4_^2−^, and BOD/COD ratio on the bacterial diversity composition of the most abundant phyla, namely, Proteobacteria, Bacteroidetes, Cloacimonetes, Cyanobacteria, Actinobacteria, Chloroflexi, Euryarchaeota, Verrucomicrobia, Firmicutes, etc. (Figure 6).

Moreover, phyla such as Bacteroidetes (r = 0.01 (BOD) and 0.11 (COD); *p*-value > 0.05), Chloroflexi (r = 0.02 (BOD) and 0.06 (COD); *p*-value > 0.05), Cloacimonetes (r = 0.39 (BOD) and 0.52 (COD); *p*-value < 0.05), Cyanobacteria (r = 0.31 (BOD) and 0.30 (COD); *p*-value < 0.05), Euryarchaeota (r = 0.08 (BOD) and 0.04 (COD); *p*-value > 0.05), and Firmicutes (r = 0.36 (BOD); *p*-value > 0.05 and 0.11 (COD); *p*-value < 0.05) show weak to strong positive correlations with environmental variables such as BOD and COD. However, some phyla, like Actinobacteria, have a strong negative significant correlation (r = −0.36 (BOD) and −0.45 (COD); *p*-value < 0.05) with BOD and COD. Other phyla, like Proteobacteria (r = −0.07 (BOD) and −0.03 (COD)), Nitrospirae (r = −0.27 (BOD) and −0.27 (COD)), and Verrucomicrobia (r = −0.12 (BOD) and −0.1 (COD)), however, have mild to weak negative correlations (*p*-value > 0.05) with these organic matter indicators. Not only that, but all phyla except Cloacimonetes (r = 0.22), Cyanobacteria (r = 0.23), Euryarchaeota (r = 0.09), and Firmicutes (r = 0.14) have a mild to weak negative non-significant (*p* value > 0.05) correlation with the BOD/COD ratio. These findings are not concurrent with the previous findings [78], where phylum Proteobacteria showed a positive correlation with BOD in the world’s largest sewage-fed farms, namely, the East Kolkata Wetlands (EKWs). This discrepancy in the results is due to the type of sewage water and differences in the treatment process; the latter mainly constitutes sewage water from domestic and industrial sites and has natural and manmade wetlands as wastewater treatment processes [78].

There is a weak to strong negative non-significant (*p*-value > 0.05) correlation between pH and the phyla Proteobacteria, Bacteroidetes, Cloacimonetes, Cyanobacteria, Chloroflexi, Euryarchaeota, Nitrospirae, and Verrucomicrobia. However, there is a significant (*p* value < 0.05) correlation between pH and Firmicutes. Additionally, phylum Actinobacteria shows a positive non-significant correlation (r = 0.15; *p*-value > 0.05). These results are aligned with the correlation between phylum Actinobacteria and pH, in contrast to the correlation between phylum Chloroflexi and the same environmental variable [76], showing a strong positive correlation between pH and both Actinobacteria and Chloroflexi. TSS, on the other hand, have a weakly positive (*p* value > 0.05) relationship with Bacteroidetes (r = 0.02), Euryarchaeota (r = 0.17), Firmicutes (r = 0.12), and Verrucomicrobia (r = 0.05). They also have a weakly negative (*p* value > 0.05) relationship with Cloacimonetes (r = −0.10), Cyanobacteria (r = −0.28), Chloroflexi (r = −0.17), Proteobacteria (r = −0.05), and Nitrospirae (r = −0.06).

Nutrients such as the nitrate (NO_3_^−^) and sulfate (SO_4_^2−^) concentrations play a pivotal role in the WWTPs. Some phyla, like Actinobacteria, Chloroflexi, Nitrospirae, and Verrucomicrobia, have a weak negative correlation with NO_3_^−^. Other phyla, like Proteobacteria, Bacteroidetes, Firmicutes, Euryarchaeota, and Cloacimonetes, have a weak positive correlation with NO_3_^−^ concentration.

There is a weak to strong positive correlation between the concentration of sulfate (SO_4_^2−^) and the phyla Actinobacteria, Chloroflexi, Nitrospirae, and Verrucomicrobia. The correlation is significant (*p*-value < 0.05) for Actinobacteria but not the other groups. Additionally, phyla such as Proteobacteria, Firmicutes, Euryarchaeota, Cyanobacteria, and Cloacimonetes have negative non-significant (*p* value > 0.05) correlations, except Firmicutes, for which it is significant.

Moreover, canonical correspondence analysis (CCA) also yields more or less similar results to the Pearson r correlation matrix. Axis 1 shows 53.64%, while axis 2 shows 30.62% bacterial diversity variation. CCA confirmed that environmental variables such as BOD, COD, BOD/COD, and nitrate (NO_3_^−^) show positive associations with phyla such as Firmicutes, Bacteroides, Cyanobacteria, Proteobacteria, Euryarchaeota, Cloacimonetes, etc. (Figure S2). On the other hand, pH and SO_4_^2−^ show weak positive and neutral correlations with most of the phyla (Figure S2).

The current dataset’s findings and significant correlations lead us to conclude that BOD and COD are the crucial environmental variables responsible for the observed narrow shifts in the community structure. The findings are in line with previous findings, where both of these environmental variables play a pivotal role in deciphering the community structure of WWTPs and other aquatic ecosystems [13,78,88].

### 3.4. Predicted Functional and Metabolic Potential of Community Structure in Pollutant Remediation

**Figure 7:**
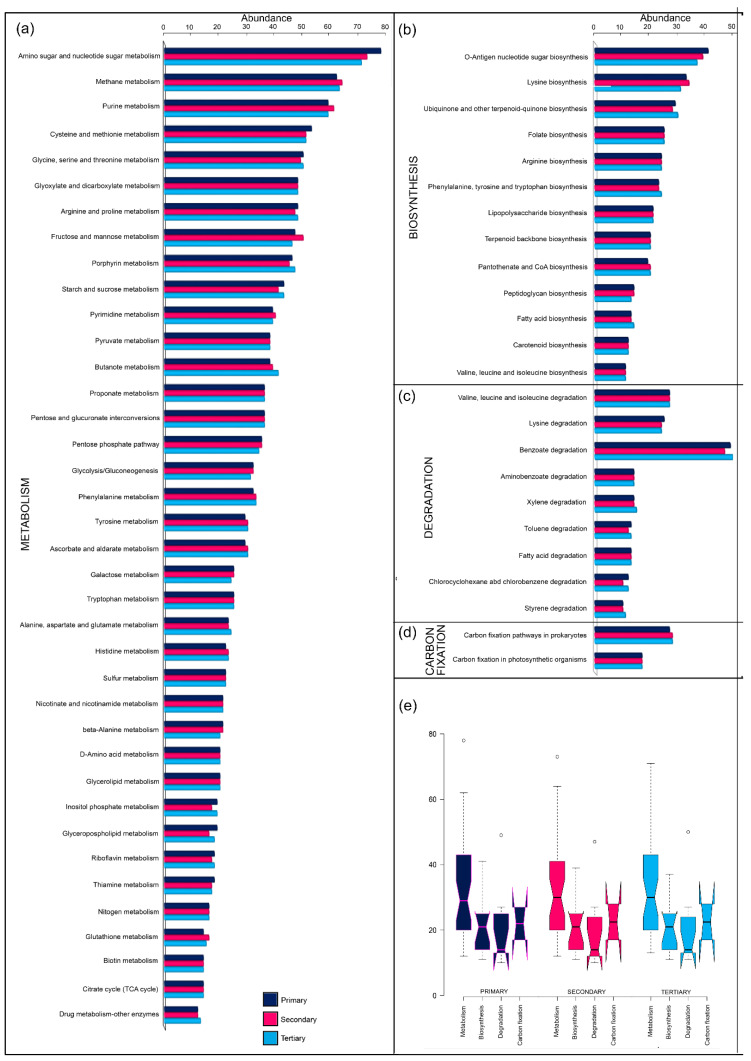
Bacterial functional annotations using PICRUSt2 analysis in different treatment stages, namely, primary, secondary, and tertiary: (**a**) Functional category (Metabolic pathways) based at Functional level 2. (**b**) Functional category (Biosynthetic pathways) based at Functional level 2. (**c**) Functional category (Degradation pathways) based at Functional level 2. (**d**) Functional category (Carbon fixation) based at Functional level 2. (**e**) Functional categories based on KEGG at Functional level 1.

The metabolic and functional potential of the abundant community structure plays a significant role in deciphering the efficiency and overall performance of the WWTPs in terms of pollutant degradation and bioremediation [13]. In the present study, the authors used the KEGG database to elucidate the metabolic and functional potential of the community structure. The findings obtained confirmed the presence of several metabolic, biosynthetic, degradation, and carbon fixation pathways (Figure 7a–e). Among these, the metabolic pathways dominated the dataset and varied in the range of ~40–75%, while on the other hand, the abundance of biosynthetic, degradation, and carbon fixation pathways varied in the ranges of ~25–40%, 25–30%, and 25–35%, respectively.

In all the treatment groups, namely, primary, secondary, and tertiary, amino acids and nucleotide sugars, followed by methane (CH₄) and the carbohydrate and nitrogen metabolic pathways, were abundant (Figure 7a). The high abundance of metabolic pathways in WWTPs signifies that the bacterial community structure is comparatively more active toward pollutant remediation through different enzymatic pathways [13,55,56]. Moreover, the functional and metabolic potential findings were in line with the roles of the abundant phyla and genera present in the current dataset, as described under Section 3.3.1, Bacterial Diversity, Composition, and Community Structure.

In terms of biosynthesis as metabolic and functional potential pathways, lysine, ubiquinone, folate, arginine, lipopolysaccharide, terpenoid, pantothenate, peptidoglycan, fatty acids, carotenoid, valine, leucine, and isoleucine dominated the dataset in all the treatment groups (Figure 7b). Members of the phylum Proteobacteria, which plays a pivotal role in the metabolic and functional utilization of amino acids, contribute significantly to the abundance of these pathways [54,55,56,57].

All the treatment groups were found to have abundant functional genes related to the degradation/decomposition of complex organic compounds such as amino acids, benzoate, aminobenzoate, xylene, toluene, fatty acids, chlorocyclohexane (C_6_H_11_Cl), chlorobenzene (C_6_H_5_Cl), styrene, etc. (Figure 7c). The current dataset confirms the significant roles of various phyla, including Proteobacteria, particularly the genus *Zavarzinia*; Bacteroidetes; Firmicutes; Cloacimonetes; and Chloroflexi, as previously described in Section 3.3.1.

Lastly, the carbon fixation pathways, equivalent in all the treatment groups (Figure 7d), were mainly attributed to the abundance of members belonging to the phylum Cyanobacteria, as they are well known for their metabolic role in inorganic carbon fixation through converting it to methane (CH₄) and generating bioenergy [8,78,79].

## 4. Future Scope in Terms of Core Pollutants Related to Sewage

Sewage contains a diverse array of toxic pollutants, including heavy metals (e.g., mercury, lead, cadmium), organic contaminants (e.g., pesticides, pharmaceuticals, endocrine-disrupting chemicals), and excess nutrients like nitrogen and phosphorus. These pollutants can exert various toxic effects, such as acute toxicity, genetic toxicity, metabolic toxicity, and inhibitory toxicity, which collectively influence microbial communities. Acute toxicity can cause immediate cell death, while genetic toxicity induces mutations in microbial DNA, potentially driving antibiotic resistance [89]. Metabolic toxicity damages enzyme pathways that are necessary for microbes to survive, and inhibitory toxicity stops growth or activity. This causes big changes in the types and numbers of microbes that live in the environment [90]. For example, toxic compounds can lower the number of sensitive species while increasing the number of resistant strains that can break down pollutants. This changes the structure of the communities and the way ecosystems work [91]. Studies that use bioassays, high-throughput sequencing, and metagenomic methods can help us understand these interactions better and make wastewater treatment processes less toxic [92,93]. In the future, studies focusing on the specific pollutants (inorganic/organic, emerging/priority, etc.) in correlation with the microbial communities’ structures and their roles in metabolism and biodegradation of pollutants is the need of the hour.

## 5. Conclusions and Recommendations in Line with SDGs

The findings suggest that a revision of the current policies or wastewater treatment protocols is necessary, with a focus on microbial treatment, particularly targeting pathogens. Even after disinfection (chloride treatment), the collected wastewater exhibits a diverse community structure, including members of the phyla Proteobacteria and Bacteroidetes. This confirms that some members of these phyla have developed resistance against chlorine, a phenomenon known as chlorine-resistant bacteria. Furthermore, the water quality of these WWTPs is poor, with parameters such as BOD, COD, and TSS violating the standard discharge norms set by the Central Pollution Control Board (CPCB), located in New Delhi, India. The current situation will serve as an alarming call for stakeholders, prompting them to take the initiative to revisit the discharge norms or enhance the facilities of the WWTPs to meet global standards. Furthermore, we recommend the implementation of online monitoring tools equipped with various sensors or the expansion of central government (CPCB) testing laboratories in partnership with state agencies such as central and state universities, state pollution control agencies, and state government-aided colleges. Moreover, clean water directly links to at least 7 SDGs, while water indirectly links to 5 SDGs. However, we specifically focus on Goal 3 and Goal 6, and we firmly believe that India must undertake infrastructural developments to meet these SDGs. This includes the establishment of new WWTPs, STPs, and ETPs; the promotion and application of constructed wetlands; and the rejuvenation of ponds and water bodies that can serve as manmade wetlands in villages for the treatment of domestic wastewater. Additionally, to manage wastewater treatment systems, an apex entity must form significant alliances with government agencies, academia, businesses, and public organizations. Communities worldwide must actively participate in updating the best management practices for WWTPs and developing innovative technological advancements for effective and efficient wastewater treatment. We firmly believe that our findings, as an interdisciplinary study that takes into account environmental variables, bacterial community structures, and their role in WWTPs, will be beneficial to potential stakeholders as they consider innovative technologies and white papers for wastewater treatment.

## Figures and Tables

**Table 1:** Water quality parameters in different treatment groups, namely, primary, secondary, and tertiary, of fourteen (14) WWTPs.

Site	Treatment Group	pH	BOD (mg/L)	COD (mg/L)	TSS (mg/L)	NO_3_^−^ (mg/L)	SO_4_^2−^ (mg/L)	BOD/COD
**Standard discharge norms (CPCB)**		5.5–9.0	10	50	20	50	150	**-**
ST 1	**PRIMARY**	6.9	85	325	102	21.5	42	0.261538
ST 2		6.9	76	330	110.2	20.1	40	0.230303
ST 3		6.9	102	340	100.3	12.1	22	0.3
ST 4		6.9	110	325	122.3	21.6	25	0.338462
ST 5		7.1	125	350	120.1	12.3	21	0.357143
ST 6		7	110	340	125.2	10.2	44	0.323529
ST 7		7.3	84	310	125	23.2	25	0.270968
ST 8		6.9	120	340	98.5	21.5	20	0.352941
ST 9		7.1	120	340	159.3	25.6	22	0.352941
ST 10		6.9	130	350	82.5	17.8	18	0.371429
ST 11		7.1	140	380	102.3	20.1	20	0.368421
ST 12		7.2	121	355	108.7	21.1	12	0.340845
ST 13		7	110	340	108.1	15.6	32	0.323529
ST 14		7.1	100	340	95.6	15.3	40	0.294118
ST 1.1	**SECONDARY**	7	80	310	98.25	18.2	40	0.258065
ST 2.1		7.3	75	328	88.36	15.2	45	0.228659
ST 3.1		7	92	325	86	8.9	44	0.283077
ST 4.1		7.1	90	310	112.3	12.8	44	0.290323
ST 5.1		7.2	90	330	116	8.6	22	0.272727
ST 6.1		7.1	105	325	120.2	8.2	32	0.323077
ST 7.1		6.9	80	300	115	12.6	12	0.266667
ST 8.1		7.1	118	325	82.3	16.2	39	0.363077
ST 9.1		8.4	105	310	141.2	22.3	40	0.33871
ST 10.1		6.9	120	355	78.1	14.2	32	0.338028
ST 11.1		7.4	135	370	100.3	18.1	32	0.364865
ST 12.1		7.3	108	325	100.5	16.4	15	0.332308
ST 13.1		7.6	110	340	104.5	8.4	44	0.323529
ST 14.1		6.9	100	300	88.4	12.6	32	0.333333
ST 1.2	**TERTIARY**	7.2	78	300	86.1	16.5	25	0.26
ST 2.2		7.3	75	320	89.25	8.2	32	0.234375
ST 3.2		7.5	92	310	85	6.3	48	0.296774
ST 4.2		7.3	89	300	105.6	10.2	49	0.296667
ST 4.3		6.9	89	300	105.6	10.2	48	0.296667
ST 5.2		7.1	86	315	115	7.2	32	0.273016
ST 6.2		7.4	101	310	116.5	6.3	15	0.325806
ST 7.2		7	75	290	98.2	10.4	48	0.258621
ST 8.2		7.1	115	315	82	13.5	36	0.365079
ST 9.2		8.9	100	300	120.3	14.1	36	0.333333
ST 10.2		7	120	345	71	11.2	40	0.347826
ST 11.2		7.8	120	365	99.8	14.3	36	0.328767
ST 12.2		8.8	104	310	98.3	11.5	40	0.335484
ST 13.2		7.5	104	325	104.1	7.6	41	0.32
ST 14.2		6.9	96	280	78.2	10.1	30	0.342857

## Data Availability

The water quality data are already included in the manuscript. Additionally, the generated raw reads were submitted to the National Center for Biotechnology Information (NCBI) database under the Bio Project id: PRJNA1011483 with accession number SAMN37220753- SAMN37220795.

## Supplementary Materials

The following supporting information can be downloaded at: https://www.mdpi.com/article/10.3390/toxics13010003/s1, Figure S1: Rarefaction analysis of wastewater samples obtained from different treatment groups namely primary, secondary and tertiary; Figure S2: Canonical correspondence analysis showing correlation between environmental variables and abundant phyla; Table S1: Details of WWTPs operational capacities; Table S2: Alpha diversity indices based on Chao1, Observed, Shannon and Simpson of wastewater water samples obtained from different treatment groups namely primary, secondary and tertiary.

## Limitations of the current work

The current works lacks the replicate dataset in terms of water quality and bacterial community structure data due to limited resources available to the authors. Additionally, one-time sampling of these WWTPs, and the failure to account for seasonal variations, could significantly impact the bacterial diversity composition and dynamics due to dilution.
