# Supplementary material for: Assessment of Bacterial Community Structure, Associated Functional Role, and Water Health in Full-Scale Municipal Wastewater Treatment Plants
Source: Toxics. 2024 Dec 24;13(1):3. doi: 10.3390/toxics13010003 (PMC11768911; doi:10.3390/toxics13010003)
Supplement: Supplementary file 1 [file toxics-13-00003-s001.zip › toxics-3364462-supplementary.pdf]

Supplementary Information

Table S1: Details of WWTPs' operational capacities.

**S. NO**	**WWTPs**	**Capacity (MLD)**	**Influent received (MLD)**	**Status**
1.	ST1	10	5.20	Working
2.	ST 2	05	3.50	Working
3.	ST 3	07	6.70	Working
4.	ST 4	11	7.40	Working
5.	ST 5	06	4.40	Working
6.	ST 6	10	4.50	Working
7.	ST 7	06	6.00	Working
8.	ST 8	06	5.80	Working
9.	ST 9	03	2.50	Working
10.	ST 10	06	6.00	Working
11.	ST 11	15	4.20	Working
12.	ST 12	07	2.00	Working
13.	ST 13	15	4.00	Working
14.	ST 14	72	50	Working
Total Capacity (MLD)		179	112.2	Current Treatment status (MLD)

Table S2. Alpha diversity indices based on Chao1, Observed, Shannon, and Simpson of wastewater water samples obtained from different treatment groups, namely primary, secondary, and tertiary.

Alpha Diversity Indices												
**Treatment Group**	**Primary**				**Secondary**				**Tertiary**			
**Samples**	**Observed**	**Chao1**	**Shannon**	**Simpson**	**Observed**	**Chao1**	**Shannon**	**Simpson**	**Observed**	**Chao1**	**Shannon**	**Simpson**
ST1	687	687.07	3.71	0.89	812	812	4.93	0.97	958	958	4.94	0.97
ST2	771	771	4.89	0.97	295	295	2.61	0.76	581	581	3.93	0.91
ST3	1770	1770	5.11	0.95	682	682	4.46	0.94	870	870	4.51	0.96
ST4	597	597	4.02	0.941	1090	1090	4.8	0.95	1388	1388	5.45	0.97
									1453	1453	6.04	0.99
ST5	634	634	4.54	0.95	435	435	3.36	0.83	569	569	4.21	0.95
ST6	906	906	5.16	0.98	688	688	3.5	0.88	194	194	3.38	0.93
ST7	1594	1594	6.37	0.99	953	953	5.59	0.99	1007	1007	4.03	0.9
ST8	1213	1213	5.81	0.99	1275	1275	5.3	0.96	1295	1295	5.78	0.98
ST9	1241	1241	5.68	0.98	1085	1085	5.56	0.98	468	468	3.9	0.9
ST10	949	949	4.82	0.96	961	961	5.29	0.98	567	567	3.59	0.88
ST11	648	648	4.84	0.97	779	779	4.43	0.93	715	715	4.4	0.95
ST12	845	845	4.53	0.95	816	816	1.35	0.32	441	441	3.08	0.8
ST13	554	554	4.9	0.98	584	584	4.06	0.93	801	801	4.87	0.98
ST14	1846	1846	6.21	0.99	1072	1072	5.14	0.97	186	186	2.4	0.76
**Mean**	1018.21	1018.21	5.04	0.96	823.35	823.35	4.31	0.885	766.2	766.2	4.30	0.92
**Standard deviation**	444.22	444.21	0.76	0.02	272.99	272.99	1.23	0.17	378.07	378.07	0.88	0.05

**Total**	1018.21 ± 444.22	1018.21 ± 444.22	5.04 ± 0.76	0.96 ± 0.02	823.35 ± 272.99	823.35 ± 272.99	4.31 ± 1.23	0.88 ± 0.17	766.2 ± 378.07	766 ± 378.07	4.30 ± 0.88	0.92 ± 0.05

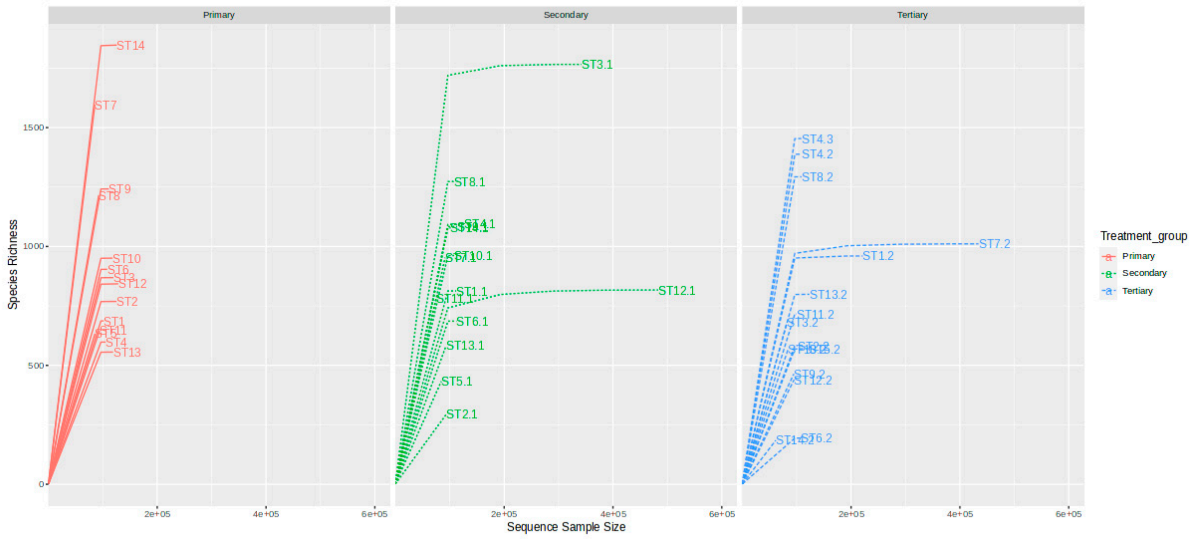

Figure S1: Rarefaction analysis of wastewater samples obtained from different treatment groups (primary, secondary, and tertiary).

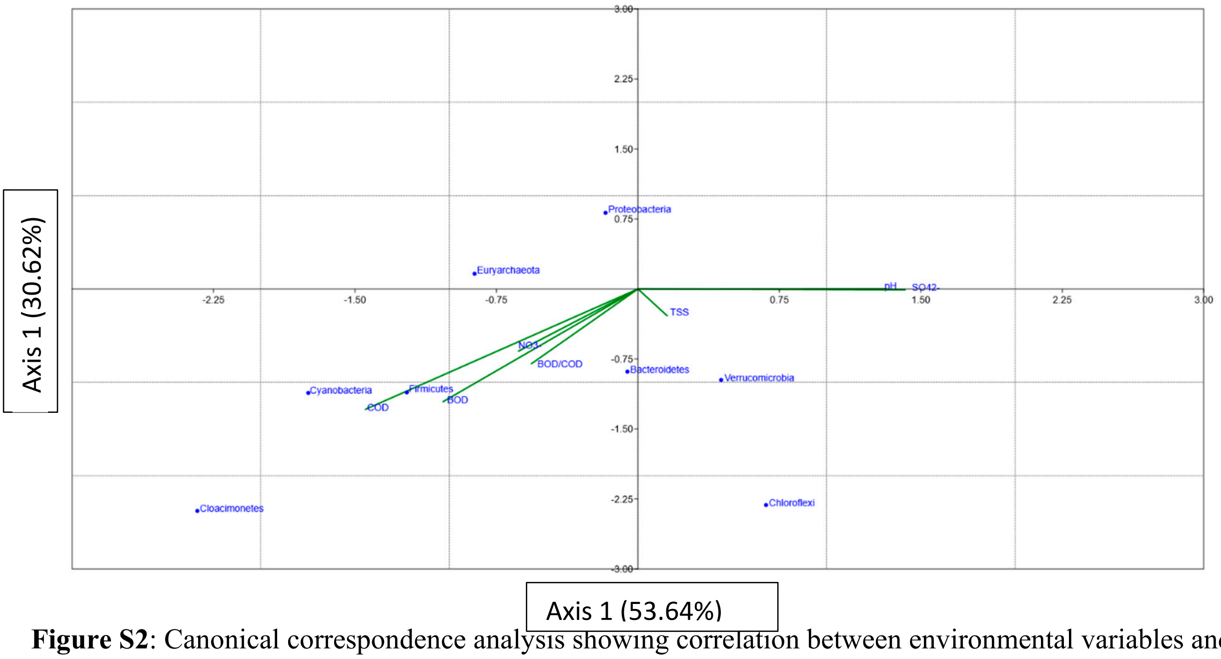

Figure S2: Canonical correspondence analysis showing correlation between environmental variables and abundant phyla

Constraints and Limitations of PICRUSt2

1. **Dependence on Reference Genomes**
   PICRUSt2 relies on a comprehensive database of reference genomes to infer gene family abundances. While the tool benefits from an extensive reference genome set, many environmental samples harbor taxa that are either underrepresented or entirely absent from these databases. This can result in reduced accuracy when predicting functional profiles, especially for unique or poorly characterized environments.
2. **Assumption of Functional Conservation**
   A core assumption in PICRUSt2 is that closely related taxa share similar functional capacities. However, microbial genomes are often subject to horizontal gene transfer, gene duplication, and gene loss, which can lead to significant variability in functional traits, even among closely related organisms. This assumption can thus compromise predictions in communities where such genomic rearrangements are common.
3. **Limitations in Detecting Novel Functions**
   The reliance on known genomes for functional inference means that PICRUSt2 may miss novel or unique functional capabilities present in a given microbial community. This issue is especially critical in ecosystems with high biodiversity or endemism, such as deep-sea vents or arctic environments.
4. **Accuracy Relative to Shotgun Metagenomics**
   Although more cost-effective, PICRUSt2 cannot match the resolution and accuracy of shotgun metagenomic sequencing, which directly measures the functional potential of microbial communities. Studies comparing these approaches have highlighted discrepancies in predicted functional pathways, especially in samples with high microbial diversity.
5. **Impact of Marker Gene Selection**
   The choice and quality of the marker gene used, typically the 16S rRNA gene, play a pivotal role in the accuracy of PICRUSt2 predictions. Variability in primer sets, amplification biases, and the resolution of marker regions can all impact the inferred functional potential, introducing biases or inaccuracies.
6. **Metadata and Environmental Context**
   PICRUSt2's predictions are not contextualized with metadata about the environment or ecological niches of the microbial community. This lack of integration can hinder the interpretation of predicted functional profiles, particularly in studies aiming to link microbial functions to environmental parameters.
7. **Computational Requirements**
   While less computationally intensive than shotgun metagenomics, running PICRUSt2 for large or complex datasets still demands significant computational resources. Researchers working with high-throughput datasets must ensure adequate infrastructure.
8. **Error Propagation**
   Errors in sequence processing, such as poor taxonomic assignments or low-quality reads, can propagate through the PICRUSt2 pipeline. This may result in highly inaccurate predictions, emphasizing the need for rigorous quality control at all preprocessing stages.
